# Hydrazine‐Assisted Acidic Water Splitting Driven by Iridium Single Atoms

**DOI:** 10.1002/advs.202305058

**Published:** 2023-09-29

**Authors:** Fang Luo, Shuyuan Pan, Yuhua Xie, Chen Li, Yingjie Yu, Haifeng Bao, Zehui Yang

**Affiliations:** ^1^ College of Materials Science and Engineering State Key Laboratory of New Textile Materials & Advanced Processing Technology Wuhan Textile University Wuhan 430200 P. R. China; ^2^ Sustainable Energy Laboratory Faculty of Materials Science and Chemistry China University of Geosciences Wuhan 388 Lumo RD Wuhan 430074 P. R. China

**Keywords:** hydrazine oxidation reaction, iridium single atoms, oxygen evolution reaction, water splitting

## Abstract

Water splitting, an efficient technology to produce purified hydrogen, normally requires high cell voltage (>1.5 V), which restricts the application of single atoms electrocatalyst in water oxidation due to the inferior stability, especially in acidic environment. Substitution of anodic oxygen evolution reaction (OER) with hydrazine oxidation reaction (HzOR) effectually reduces the overall voltage. In this work, the utilization of iridium single atom (Ir‐SA/NC) as robust hydrogen evolution reaction (HER) and HzOR electrocatalyst in 0.5 m H_2_SO_4_ electrolyte is reported. Mass activity of Ir‐SA/NC is as high as 37.02 A mg_Ir_^−1^ at overpotential of 50 mV in HER catalysis, boosted by 127‐time than Pt/C. Besides, Ir‐SA/NC requires only 0.39 V versus RHE to attain 10 mA cm^−2^ in HzOR catalysis, dramatically lower than OER (1.5 V versus RHE); importantly, a superior stability is achieved in HzOR. Moreover, the mass activity at 0.5 V versus RHE is enhanced by 83‐fold than Pt/C. The in situ Raman spectroscopy investigation suggests the HzOR pathway follows *N_2_H_4_→*2NH_2_→*2NH→2N→*N_2_→N_2_ for Ir‐SA/NC. The hydrazine assisted water splitting demands only 0.39 V to drive, 1.25 V lower than acidic water splitting.

## Graphical abstract

Ir single atom is an efficient bifunctional electrocatalyst for hydrogen evolution reaction and hydrazine oxidation reaction (HzOR) to assist acidic water splitting and in situ Raman spectroscopy reveals that HzOR on Ir single atom follows the pathway: *N_2_H_4_→*2NH_2_→*2NH→2N→*N_2_→N_2_.

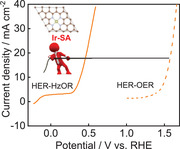

## 1 Introduction

Power to gas (P2G) is an encouraging method to store renewable energies to solve the unbalanced energy distribution. Recently, the electrochemical splitting of water to generate H_2_, with high energy density (33.6 KWh kg^−1^) superior to lithium batteries, has been reputed as one of the most exhilarating technologies for P2G.^[^ ^1^ ^]^ However, the water splitting technology is hindered by its sluggish hydrogen/oxygen evolution reaction (HER/OER) as both of them are uphill reactions demanding efficacious electrocatalyst to drive.^[^ ^2^ ^]^ Platinum (Pt) and iridium oxides (IrO_2_) is the commercially available electrocatalyst for HER and OER catalysis, respectively.^[^ ^3^ ^]^ While, the industrialization of water splitting technology encourages the development of high‐performance electrocatalyst to eliminate the utilization of noble metals.^[^ ^4^ ^]^

Recently, single atoms catalysts (SACs) have been tremendously investigated for HER and OER catalysis due to its high utilization efficiency as well as unsaturated coordination environments favorable for adsorption of reaction intermediates.^[^ ^5^ ^]^ Apart from catalytic performance, the stability of SACs should also be significantly considered, especially in OER catalysis due to the critical conditions, high voltage and strong acidic/alkaline environments leading to the easy breaking of the bond between metal and nitrogen or carbon atoms.^[^ ^6^ ^]^ In order to promote the stability of SACs in OER catalysis, rational design of support,^[^ ^7^ ^]^ modulation in coordination environments^[^ ^8^ ^]^ have been carried out. These methodologies focus on the stability of SACs in OER catalysis. Very recently, substitution of anodic OER catalysis with urea oxidation reaction (UOR),^[^ ^9^ ^]^ alcohol oxidation reaction (AOR),^[^ ^10^ ^]^ hydrazine oxidation reaction (HzOR),^[^ ^11^ ^]^ both of which possess a lower theoretical voltage compared to OER reducing the applied voltage associated with energy consumption. Among these alternative reactions, HzOR (N_2_H_4_ + 4OH^−^ → N_2_ + 4H_2_O + 4e^−^) with thermodynamical voltage of −0.33 V versus RHE has been paid much attention.^[^ ^12^ ^]^ Additionally, the hydrazine assisted water splitting has high compatibility to membrane‐free electrolyzer as N_2_ is an inert gas avoiding the mixture of H_2_/O_2_ in traditional water splitting.^[^ ^13^ ^]^ Previous studies focused on the designing the high performance electrocatalyst for HzOR in alkaline medium. As 100% atomic utilization efficiency and different local coordination condition are recorded for SACs, SACs are capable of efficiently catalyzing HzOR with relative to nanoparticle or cluster based electrocatalyst. The acidic HzOR catalysis is significantly important to alter acidic OER due to the sluggish OER kinetics in acid medium normally driven by noble metal based electrocatalyst; also, the inferior stability of acidic OER electrocatalyst caused by the high voltage leading to rapid electrochemical decomposition hinders its commercialization. SACs for HzOR catalysis in acidic electrolyte have not been reported yet.

Considering the above analysis, in this work, we report iridium single atoms as bifunctional electrocatalyst for acidic HER and HzOR catalysis to substantially lower the cell voltage for acidic water splitting. Ir‐SA/NC exhibited a robust HER performance requiring only 65 mV to reach 100 mA cm^−2^, which was lowered by 63 mV by comparison with commercial Pt/C. Also, the mass activity at overpotential of 50 mV was 37.02 A mg_Ir_^−1^ for Ir‐SA/NC, boosted by 127‐fold than commercial Pt/C. In HzOR catalysis, an 83‐time higher mass activity was also achieved for Ir‐SA/NC than Pt/C at 0.5 V versus RHE. Only 0.39 V was demanded to drive hydrazine‐assisted water splitting; therefore, a robust stability was recorded. In situ Raman spectroscopy test revealed that HzOR catalyzed by Ir‐SA/NC followed *N_2_H_4_→*2NH_2_→*2NH→2N→*N_2_→N_2_.

## 2 Results and Discussion

**Figure 1:**
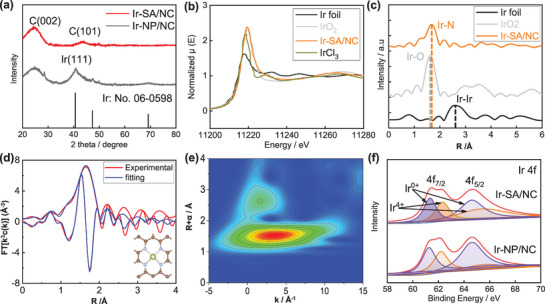
a) XRD patterns of Ir‐SA/NC and Ir‐NP/NC. b) XANES, c) EXAFS, d) fitted R space, e) WT analysis of Ir‐SA/NC. f) Ir 4f XPS spectra of Ir‐SA/NC and Ir‐NP/NC.

Prior to electrochemical test, physical characterizations have been performed. As shown in **Figure** **1**a, a broad peak at 25^o^ was observed for two electrocatalysts stemming from the (002) crystal plane of graphitic carbon. Different from Ir‐SA/NC electrocatalyst, an intensive peak at 41^o^ was detected for Ir‐NP/NC implying the formation of metallic Ir nanoparticles since this diffraction peak was assigned to (111) plane of metallic Ir according to JCPDS: PDF#06‐0598. However, this peak was vanished with the Ir loading decreasing to 0.45 wt.% confirmed by ICP‐MS. Only a weak peak centered at 43^o^ was observed due to the presence of (101) facet of graphitic carbon. The extinguished metallic Ir diffraction peak revealed the atomic dispersion of Ir in electrocatalyst. In order to confirm this point, X‐ray absorption near‐edge structure (XANES) has been tested. As shown in Figure 1b, Ir atoms in Ir‐SA/NC were in positively charged state since the white‐line intensity at Ir‐L_3_ edge was enhanced by comparison with Ir foil; also, the adsorption energy at Ir‐L_3_ edge for Ir‐SA/NC was positively shifted with relative to Ir foil.^[^ ^14^ ^]^ Thus, different local coordination condition was found in Ir‐SA/NC. As shown in Figure S1 (Supporting Information), the valance state of Ir in Ir‐SA/NC has been calculated to 2.5 based on the absorption threshold energy of Ir L_3_‐edge, in line with the XANES analysis because Ir donated its electrons to the adjacent nitrogen atoms. To deeply understand atomical coordination environment, extended X‐ray absorption fine structure (EXAFS) has been tested. As shown in Figure 1c, Ir─Ir bond was observed at 2.59 Å from Ir foil; while, Ir─Ir bond was vanished for Ir‐SA/NC and only Ir─N bond was detected at 1.68 Å since Ir─O bond centered at 1.63 Å; therefore, atomically dispersed Ir was noticed for Ir‐SA/NC and dominantly coordinated with nitrogen atoms. As shown in Figure 1d, one Ir atom coordinated with four nitrogen atoms to form Ir‐N_4_ structure was noticed in Ir‐SA/NC due to the well fitted curves. Meanwhile, the WT analysis of Ir‐SA/NC showed only one intensive peak at 3.0 Å^−1^ (Figure 1e) originating from the Ir‐N bond depicting the atomically dispersed Ir, which was different compared to Ir foil (Ir─Ir bond, Figure S2, Supporting Information). During the high temperature (950 °C), urea was decomposed to generate NH_3_; thus, nitrogen atoms were doped into carbon; simultaneously, IrCl_3_ was also decomposed and coordinated with the nitrogen atoms from nitrogen doped carbon. Ascribing to the high specific surface area of BP‐2000 (1380 m^2^ g^−1^, Figure S3, Supporting Information), plenty of active sites were capable for anchoring Ir; therefore, atomically dispersed Ir was formed with low dosage of IrCl_3_. To know the electronic configuration, XPS measurement has been conducted (Figure S4, Supporting Information). As shown in Figure 1f, the Ir atom in Ir‐SA/NC were mainly in the oxidized state by comparison with Ir‐NP/NC since the Ir atoms coordinated with nitrogen in Ir‐SA/NC and donated its electrons to nitrogen. Two peaks were found in the core‐level Ir 4f spectrum at 61.8  and 64.6 eV corresponding to Ir 4f_7/2_ and Ir 4f_5/2_ fitted to Ir^0^ and Ir^4+^ species.^[^ ^15^ ^]^ A higher valance state was observed for Ir‐SA/NC due to its atomical dispersion coordinated with nitrogen atoms. Moreover, the calculated Ir valance state from XANES was similar to the counterpart from XPS analysis. It should be noticed that oxidized Ir moieties were found for Ir‐NP/NC due to the electronic interaction between Ir‐NP and NC. As shown in Figure S5.(Supporting Information), the core level XPS spectrum of N 1s was fitted to pyridinic N, pyrrolic N, graphitic N and oxidized N moieties at 398.5, 399.9, 400.9 and 402.4 eV, respectively.^[^ ^16^ ^]^ Compared to Ir‐NP/C, the graphitic N content was decreased for Ir‐SA/NC attributing to the graphitic N atoms coordinated with Ir‐SA.^[^ ^17^ ^]^

**Figure 2:**
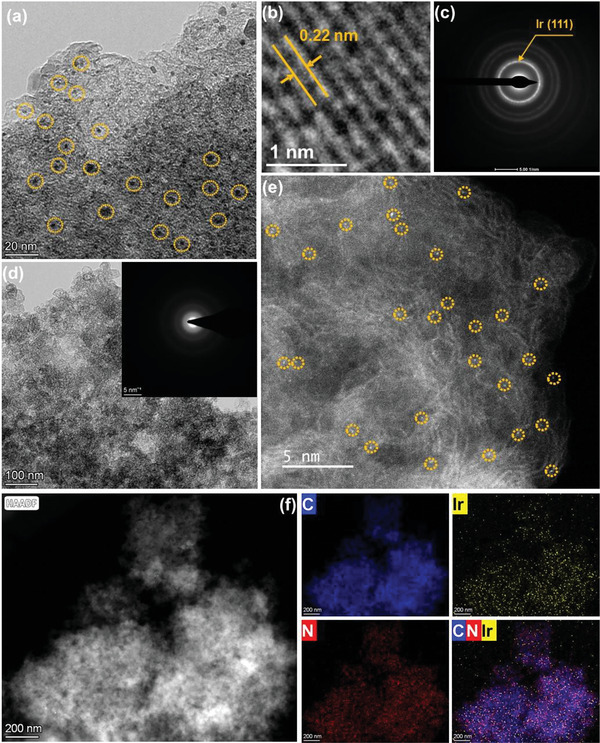
a) TEM, b) HR‐TEM, and c) SAED pattern of Ir‐NP/NC. d) TEM image and e) AC‐TEM, f) HAADF‐STEM and relative EDS mapping of Ir‐SA/NC.

The difference in morphology has been investigated by TEM. As shown in **Figure** **2**a, Ir nanoparticles were observed for Ir‐NP/NC with a diameter of 4 nm. Moreover, clear lattice spacing of 0.22 nm was detected confirming the formation of metallic Ir nanoparticle shown in Figure 2b. The SAED pattern also indicated the presence of Ir (111) plane in Ir‐NP/NC (Figure 2c). The distinct lattice spacing as well as bright ring in SAED pattern demonstrated the high crystalline of Ir‐NP/NC, well matched with XRD measurement. However, as shown in Figure 2d, nanoparticle structure was not observed. To verify the vanishing of Ir nanoparticles, SAED pattern test implied no crystal structure was formed in Ir‐SA/NC (Figure 2d). To further confirm the atomically dispersed Ir, AC‐TEM test has been performed and Ir‐SAs were distinctly observed (Figure 2e). Ir nanoparticles were still not observed in HAADF‐STEM image with a scale of 200 nm indicating the atomically dispersed Ir (Figure 2f). From the EDS mapping, it was found that Ir, N and C elements were homogenously dispersed over the electrocatalyst. To check the reproducibility, Ir‐SA/NC‐2 has been synthesized via same procedure and AC‐TEM test also verified the atomical dispersion of Ir (Figure S6, Supporting Information).

**Figure 3:**
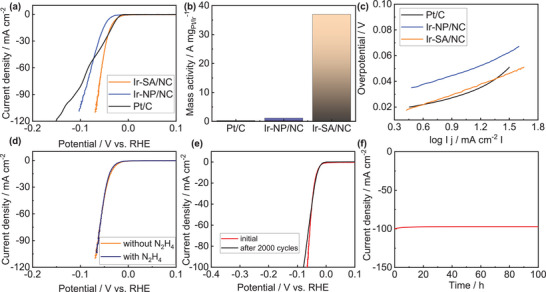
a) HER performance, b) Mass activity, and c) Tafel slope of Pt/C, Ir‐SA/NC and Ir‐NP/NC in 0.5 m H_2_SO_4_. d) Hydrazine poisoning test and e) HER cyclic stability and f) *i–t* test of Ir‐SA/NC.

First, the cathodic hydrogen evolution reaction (HER) was estimated in 0.5 m H_2_SO_4_ electrolyte. As shown in **Figure** **3**a, commercial Pt/C performed a good HER activity with overpotential of 30 and 128 mV to obtain 10 and 100 mA cm^−2^. Ir‐SA/NC exhibited a robust HER catalytic performance demanding only 31 and 65 mV to attain 10 and 100 mA cm^−2^, decreased by 16 and 32 mV than Ir‐NP/NC due to the atomical dispersion of Ir leading to a high utilization efficiency associated with different coordination environments (Ir─N vs Ir─Ir). Moreover, the HER onset potential was as low as 6 mV for Ir‐SA/NC, lowered by 10 and 17 mV than Pt/C and Ir‐NP/NC. Additionally, the overpotential at 100 mA cm^−2^ was largely decreased by 63 mV by comparison with Pt/C revealing the better HER performance of Ir‐SA/NC. As the noble metal loading was different, mass activity was calculation and Ir‐SA/NC reached 37.02 A mg_Ir_^−1^, boosted by 32‐ and 127‐fold than Ir‐NP/NC and Pt/C at overpotential of 50 mV (Figure 3b) underscoring the superior catalytic performance of Ir‐SAs. To quantify the electrochemical surface area (ESA), double layer capacitance, proportionating to ESA, has been evaluated from cyclic voltammetry curve.^[^ ^18^ ^]^ As shown in Figure S7 (Supporting Information), *C*_dl_ of Ir‐SA/NC was 39.9 mF cm^−2^, boosted by 23 times than Ir‐NP/NC as all Ir atoms were active during HER catalysis. The electrochemical surface area (ECSA) was evaluated from the equation: ECSA = *C*_dl_/*C*_s_ (*C*_s_ represents the specific capacitance); therefore, ECSA was 350 and 15.2 m^2^ g^−1^ for Ir‐SA/NC and Ir‐NP/NC, respectively. The larger ECSA of Ir‐SA/NC highlighted the higher atomic utilization efficiency of SACs. Additionally, as shown in Figure S8 (Supporting Information), charge transfer resistance (*R*_ct_) was 65 Ω and 100 Ω for Ir‐SA/NC and Ir‐NP/NC, respectively. The Tafel slope, descriptor of reaction kinetics, was 29.7, 33.0, and 35.9 mV dec^−1^ for Ir‐SA/NC, Ir‐NP/NC and Pt/C, respectively (Figure 3c). The lower Tafel slope indicated a faster HER kinetics of Ir‐SA/NC. As the theoretical Tafel slope is 120, 40, and 30 mV dec^−1^ for Volmer, Heyrovsky, and Tafel step in acidic HER catalysis, respectively. Pt/C and Ir‐NP/NC followed a Volmer–Heyrovsky HER mechanism; while, Volmer–Tafel mechanism was noticed for Ir‐SA/NC.^[^ ^19^ ^]^ To assess the intrinsic activity, turnover frequency was calculated and Ir‐SA/NC reached 33.3 s^−1^ at overpotential of 50 mV, which was increased by a factor of 31 and 61 than Ir‐NP/NC and Pt/C (Figure S9, Supporting Information). The exchange current density, fingerprint of intrinsic catalytic performance, was estimated to 0.56 mA cm^−2^, boosted by 5.1‐ and 1.5‐fold than Ir‐NP/NC (0.11 mA cm^−2^) and Pt/C (0.37 mA cm^−2^) highlighting the better intrinsic HER activity. Ascribing to the N_2_H_4_‐assited water splitting, the affection of N_2_H_4_ in HER performance was also estimated. As shown in Figure 3d, inconspicuous decay in HER performance was observed with the presence of N_2_H_4_ in electrolyte. The stability was tested with potential cycling from −0.2 to 0.2 V versus RHE. As shown in Figure 3e, a stable HER performance was recorded for Ir‐SA/NC and overpotential at 100 mA cm^−2^ was only deteriorated by 14 mV after 2000 cycles. Also, the Tafel slope was 30.2 mV dec^−1^ for Ir‐SA/NC after stability test highlighting the superior stability of Ir‐SA/NC (Figure S10, Supporting Information). To know the change in electrochemical property, *C*_dl_ was estimated to 37.2 mF cm^−2^ after 2000 CV cycles (Figure S11, Supporting Information) decayed by only 6%. Furthermore, the *R*_ct_ was merely increased by 15% demonstrating the well‐preserved structure during potential cycling (Figure S12, Supporting Information). However, commercial Pt/C performed a lower stability as overpotential at 10 mA cm^−2^ was increased by 16 mV after 2000 cycles (Figure S13, Supporting Information) evidenced by the decrement in C_dl_. As shown in Figure 3f, Ir‐SA/NC maintained its HER activity of 100 mA cm^−2^ during 100 h long‐term catalysis revealing its superior stability. HER performance of Ir‐SA/NC showed an ignorable decay after CA test (Figure S14, Supporting Information) revealing the high stability of Ir‐SA/NC during HER catalysis.

**Figure 4:**
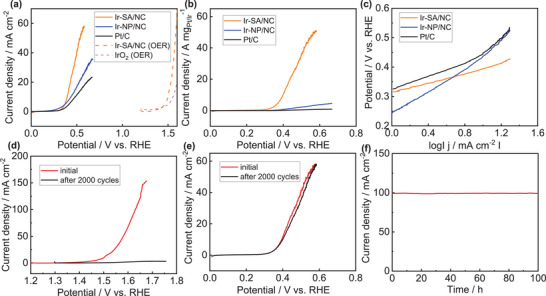
a) HzOR performance, b) Mass activity, and c) Tafel slope of Pt/C, Ir‐SA/NC and Ir‐NP/NC in 0.5 m H_2_SO_4_. d) OER and e) HzOR cyclic stability and f) *i–t* test of Ir‐SA/NC.

As shown in **Figure** **4**a, the anodic reaction was also tested. Ascribing to the atomically dispersed Ir, Ir‐SA/NC performed an exceptional hydrazine oxidation reaction (HzOR) requiring 0.39 V versus RHE to achieve 10 mA cm^−2^ in acidic medium, which was lower than Ir‐NP/NC (0.44 V vs RHE) and Pt/C (0.48 V vs RHE). Besides, the potential was significantly decreased for Ir‐SA/NC with relative to Ir‐NP/NC and Pt/C. Also, the oxygen reduction reaction (OER) performance of Ir‐SA/NC was estimated demanding overpotential of 270 mV to reach 10 mA cm^−2^, a superior OER catalytic activity; while the potential was decreased by 1.11 V by comparison with HzOR. The lower potential was beneficial for stability since breaking Ir─N bond was more susceptible to occur at high potential in 0.5 m H_2_SO_4_. Also, compared to the acidic OER catalysis driven by commercial IrO_2_, the substitution of HzOR by Ir‐SA/NC lowered the potential by 1.17 V demonstrating the superiority of HzOR catalysis. As shown in Figure 4b, the mass activity of Ir‐SA/NC at 0.5 V versus RHE was 36.3 A mg_Ir_^−1^, boosted by 17‐ and 83‐time than Ir‐NP/NC and Pt/C. As displayed in Table S1 (Supporting Information), Ir‐SA/NC has been recognized as one of the most efficient HzOR electrocatalysts. To prove the high catalytic performance, *C*_dl_ was also monitored.^[^ ^20^ ^]^ As shown in Figure S15 (Supporting Information), Ir‐SA/NC showed a 4.7‐time higher *C*_dl_ than Ir‐NP/NC. Additionally, a lower R_ct_ was also noticed for Ir‐SA/NC (Figure S16, Supporting Information). To know the HzOR kinetics, Tafel slope was plotted in Figure 4c, in which Tafel slope was 78.0, 190.0, and 114.0 mV dec^−1^ for Ir‐SA/NC, Ir‐NP/NC and Pt/C, respectively. The lower Tafel slope suggested N_2_H_4_ molecule was prone to oxidize on Ir‐SA/NC. Ir‐SA/NC exhibited an exceptional OER performance; nevertheless, as shown in Figure 4d, the OER performance was dramatically deteriorated ascribing to the harsh condition decomposing Ir‐SA/NC since the electronic coupling strength was not enough to resist electrochemical dissolution. Interestingly, as shown in Figure 4e, the potential at 50 mA cm^−2^ was only increased by 12 mV in HzOR catalysis due to the comparably lower applied potential. The similar Tafel slope obtained after 2000 cycles also indicated the superior stability of Ir‐SA/NC in HzOR catalysis (Figure S17, Supporting Information). As shown in Figure S18 (Supporting Information), *C*_dl_ was only degraded by 8% after 2000 cycles implying the excellent structural stability. A similar *R*_ct_ was observed for Ir‐SA/NC before and after potential cycling (Figure S19, Supporting Information). The HzOR activity of Ir‐SA/NC was stable at 100 mA cm^−2^ in 100 h (Figure 4f) underlining the excellent structural stability due to the low applied potential by comparison with OER catalysis. As shown in Figure S20 (Supporting Information), a similar HzOR performance was recorded for Ir‐SA/NC before and after CA test indicating the superb structural stability in HzOR catalysis. As shown in Figure S21 (Supporting Information), Ir 4f and N 1s peaks were both detected after *i–t* test for Ir‐SA/NC. Moreover, the deconvoluted Ir 4f peak revealed a slight increment in percentage of oxidized Ir species underscoring the superior structural stability of Ir‐SA/NC during HzOR catalysis.

**Figure 5:**
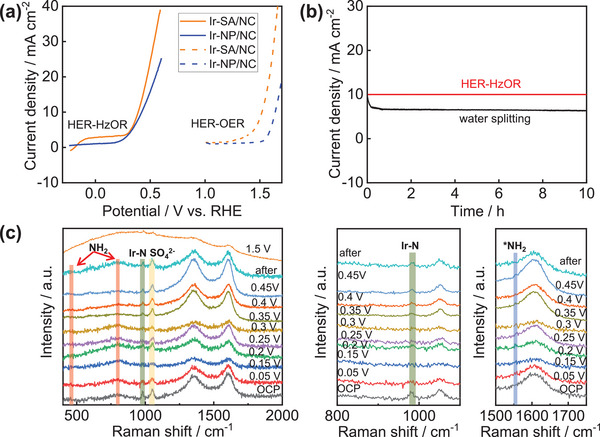
a) Hydrazssine assist‐ and overall water splitting performance of Ir‐SA/NC and Ir‐NP/NC. b) HER‐HzOR and HER‐OER stability of Ir‐SA/NC. c) In situ Raman spectroscopy of Ir‐SA/NC.

As shown in **Figure** **5**a, Ir‐SA/NC required only 0.39 V to reach 10 mA cm^−2^ in HER‐HzOR catalysis, 40 mV lower than Ir‐NP/NC. Meanwhile, Ir‐SA/NC showed a better water splitting (HER‐OER) performance than Ir‐NP/NC due to the high catalytic performance of Ir‐SAs. Compared to water splitting, HER‐HzOR catalysis efficiently reduced the applied potential by 1.25 V, which could be favorable for the stability of Ir‐SAs in acidic medium. As shown in Figure 5b, a stable current density (100 mA cm^−2^) was recorded for HER‐HzOR coupled catalysis driven by Ir‐SA/NC during 100 h suggesting the potential unitization of Ir‐SA/NC in practical application of N_2_H_4_ assisted water splitting. While the current density was sharply decayed due to the high potential causing the electrochemical decomposition of Ir‐SA/NC. As two possible HzOR pathways were recognized, in situ Raman spectroscopy has been performed to determine. As shown in Figure 5c, D and G bands from carbon were clearly observed due to the utilization of NC as support; moreover, the D/G ratio (0.9) was almost consistent with potential increased to 0.25 V versus RHE; while, D/G ratio was sharply increased to 1.2 due to the serious electrochemical oxidization of carbon triggering the loss of Ir‐SAs; thereby, Ir‐SA/NC exhibited an inferior stability in OER catalysis.^[^ ^21^ ^]^ Raman peaks at 485 and 779 cm^−1^ were assigned to the NH_2_ pitching and NH_2_ antisymmetric deformation ascribing to the adsorption of N_2_H_4_ on electrocatalyst.^[^ ^22^ ^]^ Peak at 972 cm^−1^ was the signal from Ir─N structure.^[^ ^23^ ^]^ An intensive peak at 1056 cm^−1^ was corresponding to the symmetric stretching vibrational mode of SO_4_^2−^ demonstrating the good wettability of Ir‐SA/NC.^[^ ^24^ ^]^ Interestingly, with applied potential, a shoulder peak at 1568 cm^−1^ was emerged N─H bending mode of *NH_2_ intermediates and vanished after HzOR catalysis, which was a strong indication of HzOR pathway via *N_2_H_4_→*2NH_2_→*2NH→2N→*N_2_→N_2_.^[^ ^25^ ^]^

**Figure 6:**
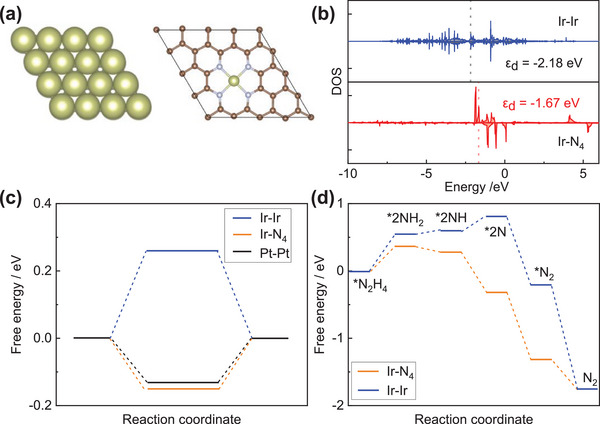
a) Chemical structures, b) density of state, c) Gibbs free energy for hydrogen, and d) free energies in HzOR catalysis of Pt (111), Ir (111), and Ir‐N_4_ structures.

Theoretical calculation was invited to deeply understand the mechanism at atomic level. As shown in **Figure** **6**a, chemical structure of metallic Ir and Ir‐N_4_ were established based on the EXAFS result. First, the density of state (DOS) has been calculated. The d band center of Ir in Ir‐N_4_ structure was −1.67 eV (Figure 6b), upshifted to Femi level compared to metallic Ir (−2.18 eV) promoting the electro‐adsorption of reaction intermediates. As shown in Figure 6c, Gibbs free energy for hydrogen (ΔG_H*_) was invited as descriptor of HER performance.^[^ ^26^ ^]^ Ir─Ir structure showed a ΔG_H*_ of 0.26 eV implying the difficulty in adsorption of hydrogen atoms; while, Ir‐N_4_ performed a Δ*G*_H*_ of −0.15 eV, closer to 0 eV than Ir─Ir suggesting the better HER performance. Furthermore, the free energy of HzOR catalysis was also calculated. As shown in Figure 6d, the primary dehydrogenation of N_2_H_4_ molecule was recognized as rate‐limiting step. Ir‐N_4_ structure required only 0.37 eV, which was 0.18 eV lower than Ir─Ir revealing the better HzOR catalytic capability.

## 3 Conclusion

In summary, we have constructed iridium single atom electrocatalyst via a high‐temperature pyrolysis method. The EXAFS result suggested the formation of Ir‐N_4_ structure. The HER test indicated Ir‐SA/NC exhibited a robust performance with overpotential of 50 mV to reach 52 mA cm^−2^, equal to a 127‐fold higher mass activity than Pt/C in acidic medium. Moreover, the substitution of OER with HzOR, the potential to reach 10 mA cm^−2^ was reduced by 1.11 V. Due to the decreased potential, a high stability was achieved. The cell voltage for overall water splitting was efficiently lowered by 1.25 V for Ir‐SA/NC with Hydrazine assistance. The in situ Raman spectroscopy revealed that NH_2_ species were generated during HzOR; thereby, HzOR catalysis followed the pathway: *N_2_H_4_→*2NH_2_→*2NH→2N→*N_2_→N_2_ for Ir‐SA/NC.

## 4 Experimental Section

### Materials

Melamine, ethyl alcohol, and hydrochloric acid (32–37 wt.%) were purchased from Sinopharm Chemical Reagent Co., Ltd. Iridium (III) trichloride (IrCl_3_, 99.95%) and iridium (IV) oxide (IrO_2_, 99.9%) were purchased from Aldrich Chemical. Nafion solution (5 wt.%) and commercial Pt/C (Pt amount: 20 wt.%) were obtained from Alfa Aesar. All the chemicals were used as received without any purification. The water used in all experiments was deionized water.

### Synthesis of Electrocatalyst

For the synthesis of Ir‐SA/NC electrocatalyst, 100 mg of BP2000 and 0.9 mg of IrCl_3_ were dispersed in 6 m HNO_3_ (30 mL). The mixture was magnetically stirred under 80 °C in an oil bath for 6 h, then dried at 60 °C for 10 h. Mixing the resultant powder with 1 g of urea in an agate mortar, the mixture was transferred to a ceramic boat and calcined in a tube furnace at 950 °C for 1 h under nitrogen atmosphere with the flow rate of 5 °C min^−1^. After that, Ir‐SA/NC electrocatalyst was obtained. For Ir‐NP/NC synthesis, 9 mg IrCl_3_ was utilized.

### Material Characterization

The crystal structure of the synthesized electrocatalyst was detected by X‐ray diffraction (XRD, Bruker AXS D8‐Focus, Germany) with Cu Kα radiation in the range of 2*θ* from 30° to 70°. The X‐ray photoelectron spectroscopy (XPS) spectra were measured using Thermo‐Scientific K‐*α* equipment. SEM images were recorded by Hitachi SU8010. HAADF‐STEM and EDS mappings were conducted with a FEI Tecnai G2 F20 electron microscope with accelerating voltage of 200 KV. The Ir L_3_‐edge XAS spectra was carried in fluorescence mode in SPring‐8. The acquired XAS data were processed according to the standard procedures using the ATHENA module implemented in the IFEFFIT software package and FEFF8.20.

### Electrochemical Measurements

All the electrochemical tests were carried out at room temperature of 25 °C using Gamry (interface 1000E, USA) instrument with a typical three‐electrode system. In a typical preparation procedure of working electrode, 2 mg electrocatalysts were dispersed in 800 µL of deionized water, 185 µL of isopropanol and 15 µL of Nafion solution (5 wt.%) by ultrasonic treatment for 30 min to obtain the homogeneous suspension. Then the ink was cast on a glassy carbon electrode (GCE, 3 mm in diameter) to prepare a working electrode. The 0.5 m H_2_SO_4_ solution was used for the electrochemical electrolyte and the solutions were saturated with N_2_ for 30 min prior to the HER or overall water splitting test or with O_2_ prior to the OER/HzOR test. For HER test, an Ag/AgCl electrode saturated with KCl, and a carbon rod were used as the reference and counter electrodes, respectively. LSV curve was recorded with a scan rate of 5 mV s^−1^. CV was carried out with 50 mV s^−1^ ranging from 0 to 0.5 V versus RHE. The electrochemical double‐layer capacitance was calculated from the cyclic voltammetry curves recorded from 0.16 to 0.26 V versus RHE with different scan rates from 10 to 100 mV s^−1^ in HER catalysis. EIS was measured from 100 kHz to 0.05 Hz under AC voltage amplitude of 5 mV and DC voltage based at a given potential at 10 mA cm^−2^. The long‐term stability was measured by chronoamperometric (*i–t*) stability examination at a given potential. Oxygen evolution reaction (OER) test was carried out in O_2_‐saturated 0.5 m H_2_SO_4_ solution. Hydrazine oxidation reaction (HzOR) was tested in O_2_‐saturated 0.5 m H_2_SO_4_ solution with 0.33 m N_2_H_4_. The overall water splitting test was performed in 0.5 m H_2_SO_4_ solution using catalyst ink cast on GCE as both cathode and anode in a two‐electrode system with catalyst loading of 0.285 mg cm^2^. Turnover frequency (TOF) was calculated based on the following equation:

(1) $$\begin{array}{ccc} & & {\mathrm{TOF}}_{\mathrm{catalyst}}\left(s^{-1}\right)=i_{0}\left({A\;\mathrm{cm}}^{-2}\right)\\ & & \;/\left\{\left[\mathrm{Density}\;\mathrm{of}\;\mathrm{Ir}\;\mathrm{atoms}\;\mathrm{in}\;\mathrm{the}\;\mathrm{catalyst}\left(\mathrm{sites}/{\mathrm{cm}}^{2}\right)\right]\right.\\ & & \left.\times \left[1.602x10^{-19}\left(C/e^{-}\right)\right]\;x\;\left[2e^{-}/H_{2}\right]\right\} \end{array}$$

### Computational Details

All periodic density functional theory (DFT) calculations with spin polarization were performed by using the Vienna ab initio simulation package (VASP) with the Perdew–Burke–Ernzerhof (PBE) exchange‐correlation functional. The projector‐augmented plane wave (PAW) was used to describe the interactions between core electrons and ions. A plane‐wave cutoff energy was tested and set to 400 eV in all calculations. 3 × 3 × 1 Monkhorst‐Pack grid k‐points are employed for geometric optimization, and the convergence threshold is set as 10^−4^ eV in energy and 0.02 eV Å^−1^ in force, respectively. Ir‐SA/NC and Ir─Ir models were employed. A vacuum distance of 15 Å was imposed between neighboring slab images in order to avoid interactions between periodic images. It should be noted that the convergence threshold was set to 10^−7^ eV during calculation.

### Hydrogen Evolution Reaction

The hydrogen adsorption free energies were calculated by the following equation: Δ*G*_H*_ = Δ*E*_H*_ + Δ*ZPE − T*Δ*S*, where Δ*E*_H*_ is the hydrogen chemisorption energy. ΔZPE and ΔS are the zero‐point energy difference and the entropy difference between the adsorbed and the gas phase, respectively. Here, the hydrogen chemisorption energy was defined by Δ*E*_H*_ = 1/n (*E*(slab+nH) − *E*(Surf) − n/2 *E*(H_2_)), where *n* is the number of H atoms in the calculations, *E*(slab+nH), *E*(Surf), and *E*(H_2_) are the total energies of the adsorption of n H atoms, clean surfaces, and gaseous hydrogen molecule, respectively.

## Conflict of Interest

The authors declare no conflict of interest.

## Supporting information

Supporting Information

## Data Availability

The data that support the findings of this study are available from the corresponding author upon reasonable request.
