# Supplementary material for: Hydrazine‐Assisted Acidic Water Splitting Driven by Iridium Single Atoms
Source: Adv Sci (Weinh). 2023 Sep 29;10(32):2305058. doi: 10.1002/advs.202305058 (PMC10646228; doi:10.1002/advs.202305058)
Supplement: Supplementary file 1 — Supporting Information [file ADVS-10-2305058-s001.pdf]

ADVANCED SCIENCE
Open Access

Supporting Information

for *Adv. Sci.*, DOI 10.1002/advs.202305058

Hydrazine-Assisted Acidic Water Splitting Driven by Iridium Single Atoms

Fang Luo, Shuyuan Pan, Yuhua Xie, Chen Li, Yingjie Yu, Haifeng Bao* and Zehui Yang**

Supporting Information

Hydrazine-assisted acidic water splitting driven by iridium single atoms

Fang Luo[a#], Shuyuan Pan[b#], Yuhua Xie[b], Chen Li[a], Yingjie Yu[a], Haifeng Bao[a*] and Zehui Yang[b*]*

Table S1 Comparison of HzOR catalytic performance of recently reported nobel metal based electrocatalysts.

**Electrocatalyst**	**Electrolyte**	**Mass activity@0.2 V vs. RHE**	**Refs.**
Ir-SA/NC	0.5 M H_2SO_4+0.33 M N_2H_4	8.61 A mg_{Ir}^{-1}	This work
Ru/NSCS	1 M KOH+0.4 M N_2H_4	7.65 A mg_{Ru}^{-1}	S1
RuPd/C	1 M KOH+0.5 M N_2H_4	2.21 A mg_{Ru+Pd}^{-1}	S2
Vo-WO_3/Ru SAs	1 M PBS+0.5 M N_2H_4	5.00 A mg_{Ru}^{-1}	S3
Ru SA/NC	1 M KOH+0.5 M N_2H_4	1.20 A mg_{Ru}^{-1}	S4
RhIr	1 M KOH+0.3 M N_2H_4	1.93 A mg_{Rh+Ir}^{-1}	S5
Ru SA/NC	1 M KOH+0.4 M N_2H_4	0.94 A mg_{Ru}^{-1}	S6
Ir/PNC	1 M KOH+0.5 M N_2H_4	0.63 A mg_{Ir}^{-1}	S7
Ru/NC	0.5 M H_2SO_4+0.05 M N_2H_4	0.01 A mg_{Rh}^{-1}	S8
Ru-NSs-300	1 M KOH+0.5 M N_2H_4	0.38 A mg_{Ru}^{-1}	S9
RhPb NFs	1 M KOH+0.1 M N_2H_4	1.41 A mg_{Rh}^{-1}	S10

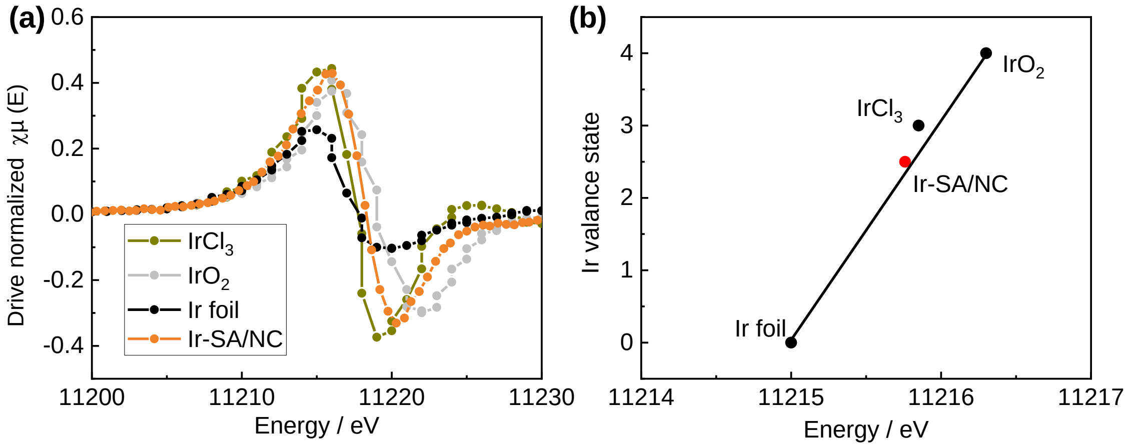

Figure S1 (a) Absorption threshold energy of Ir L_3-edge of various electrocatalysts. (b) Calculated valence states of Ir electrocatalysts.

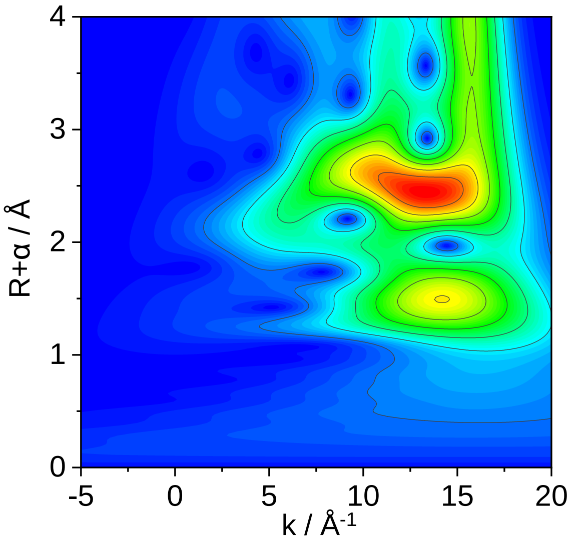

Figure S2 WT analysis of Ir foil.

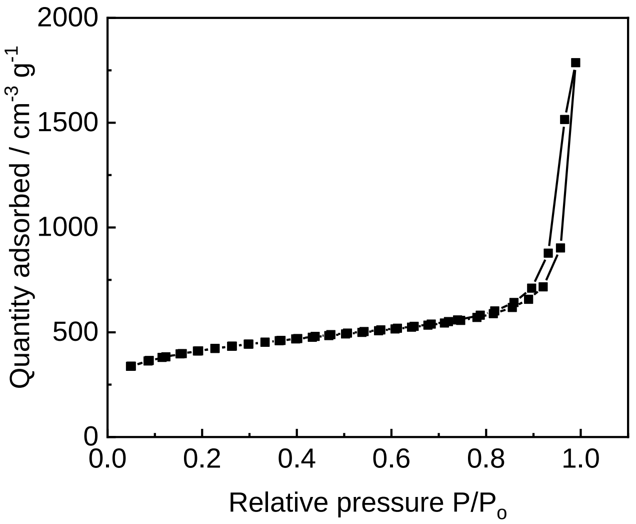

Figure S3 N_2 isothermal curves of BP-2000.

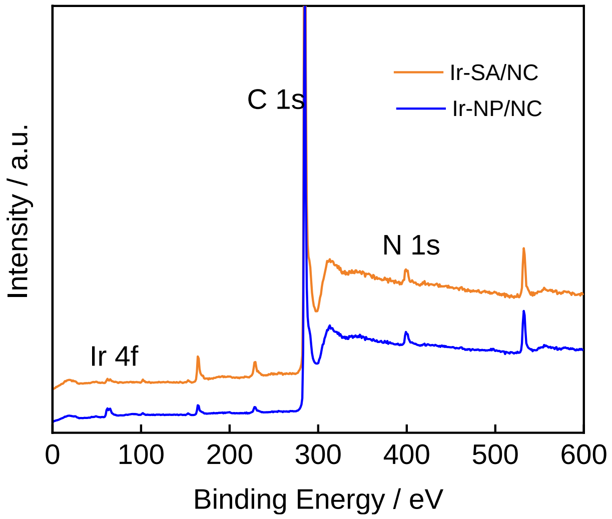

Figure S4 XPS survey scan of Ir-SA/NC and Ir-NP/NC electrocatalysts.

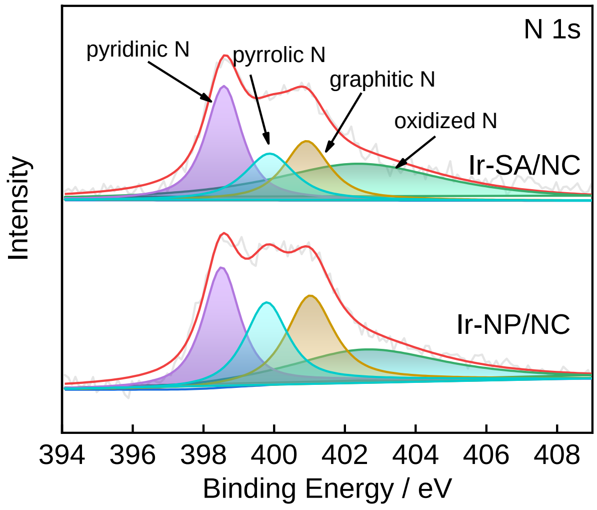

Figure S5 N 1s peaks of Ir-SA/NC and Ir-NP/NC electrocatalysts.

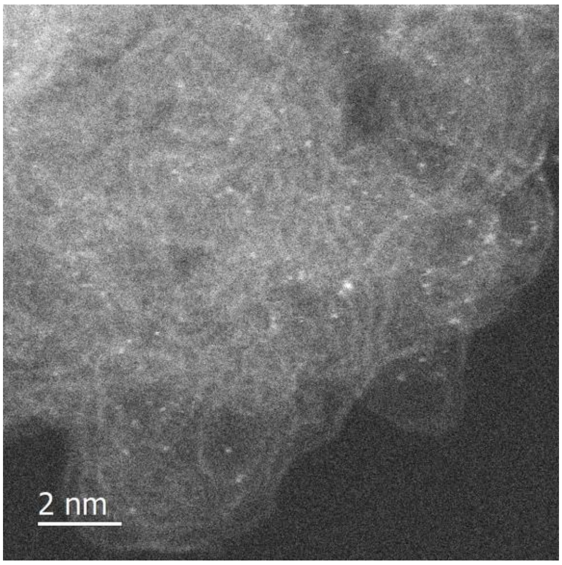

Figure S6 AC-TEM image of Ir-SA/NC-2 electrocatalyst.

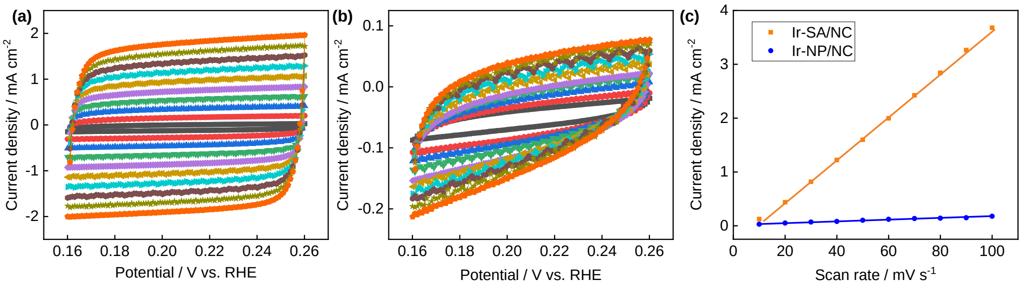

Figure S7 Cyclic voltammetry curves of Ir-SA/NC (a) and Ir-NP/NC (b) electrocatalysts. (c) C_{dl} of Ir-SA/NC and Ir-NP/NC electrocatalysts.

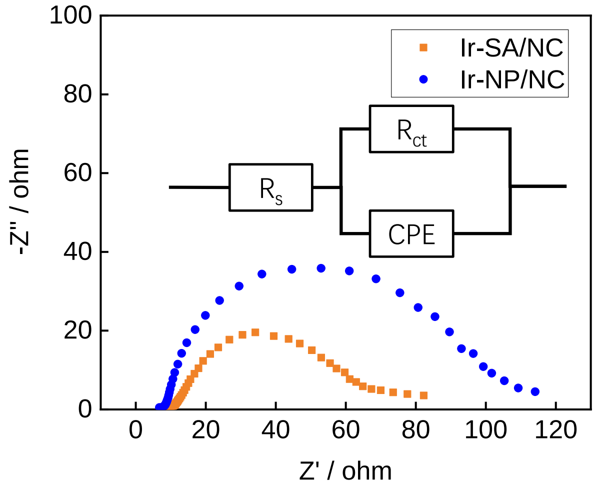

Figure S8 Electrochemical impedance spectroscopies of Ir-SA/NC and Ir-NP/NC electrocatalysts.

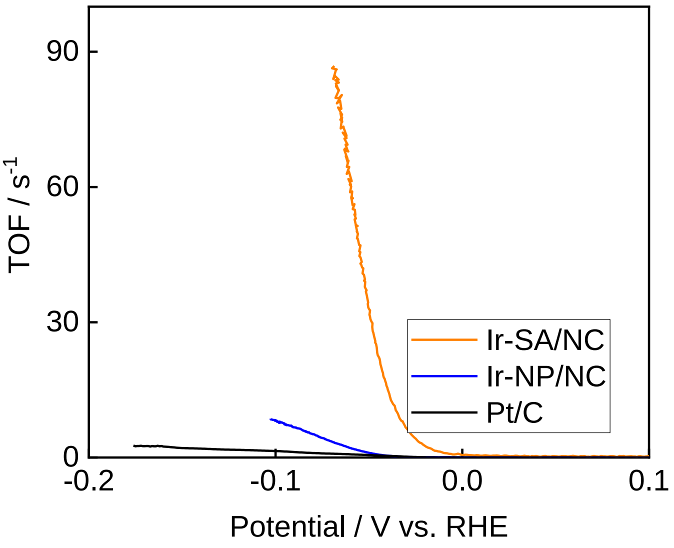

Figure S9 Turnover of frequency of Pt/C, Ir-SA/NC and Ir-NP/NC electrocatalysts.

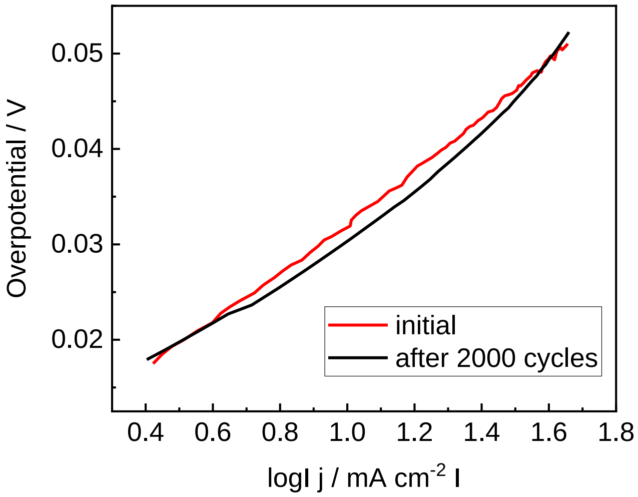

Figure S10 Tafel slopes of Ir-SA/NC electrocatalyst before and after 2000 cycles.

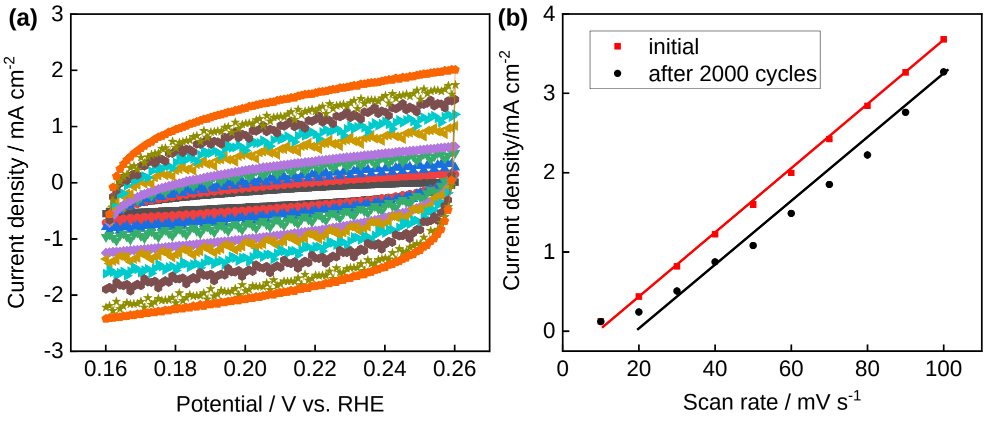

Figure S11 (a) Cyclic voltammetry curve of Ir-SA/NC after 2000 cycles. (b) C_{dl} of Ir-SA/NC before and after 2000 cycles.

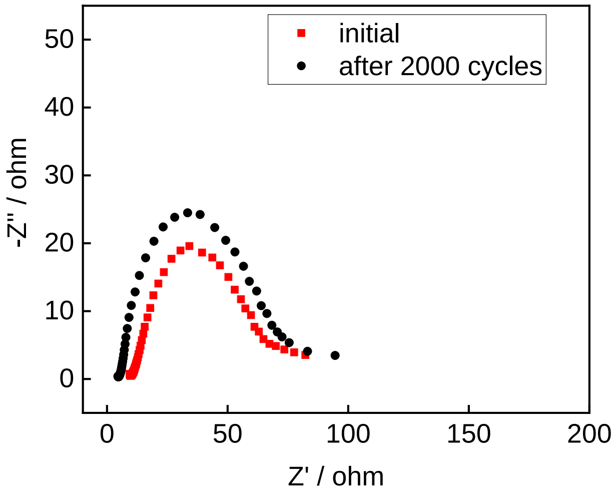

Figure S12 Electrochemical impedance spectroscopies of Ir-SA/NC before and after 2000 cycles.

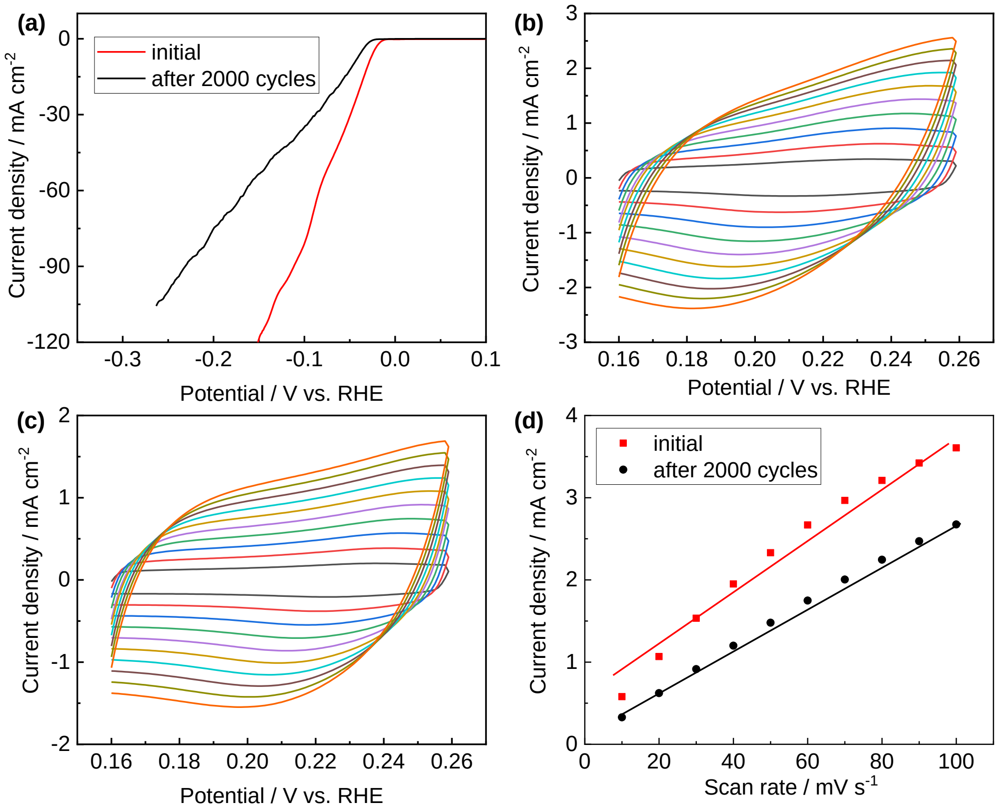

Figure S13 HER performance (a), cyclic voltammetry (b, c) curves and C_{dl} (d) of commercial Pt/C before and after 2000 cycles.

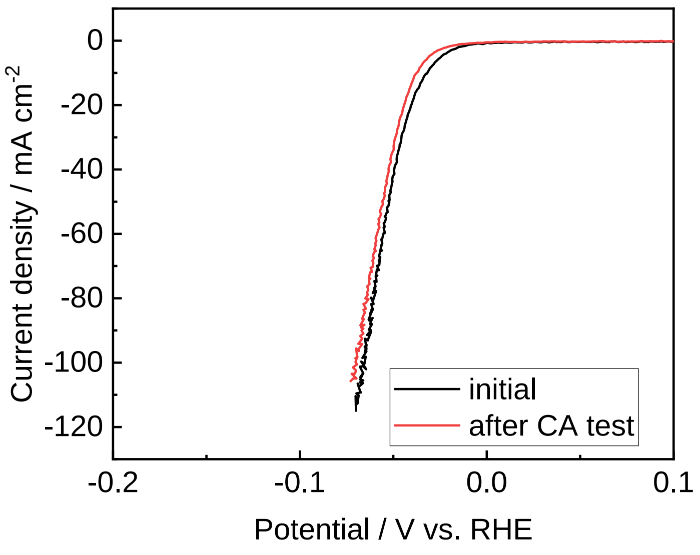

Figure S14 HER perfomrance of Ir-SA/NC before and after CA test.

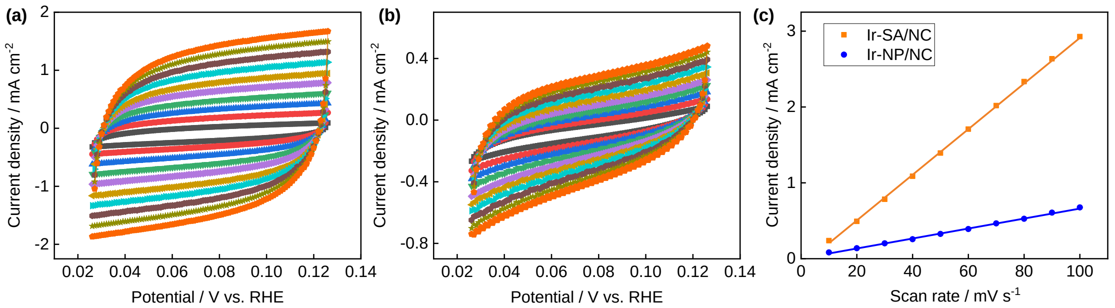

Figure S15 Cyclic voltammetry curves of Ir-SA/NC (a) and Ir-NP/NC (b) electrocatalysts. (c) C_{dl} of Ir-SA/NC and Ir-NP/NC electrocatalysts.

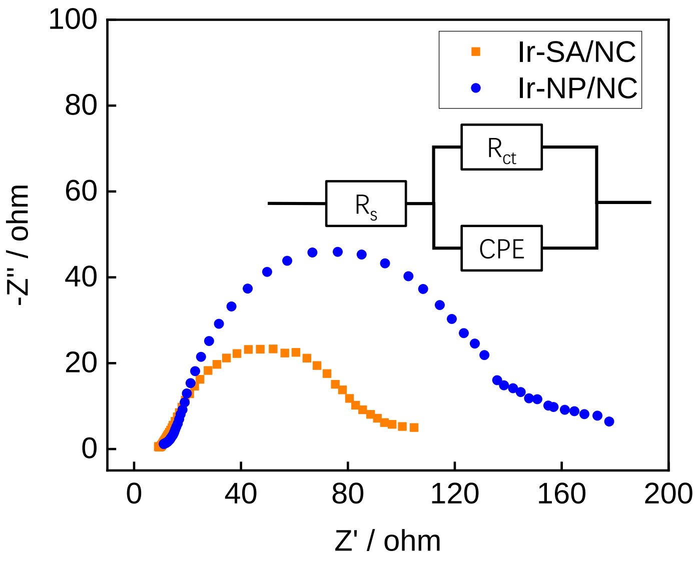

Figure S16 Electrochemical impedance spectroscopies of Ir-SA/NC and Ir-NP/NC electrocatalysts.

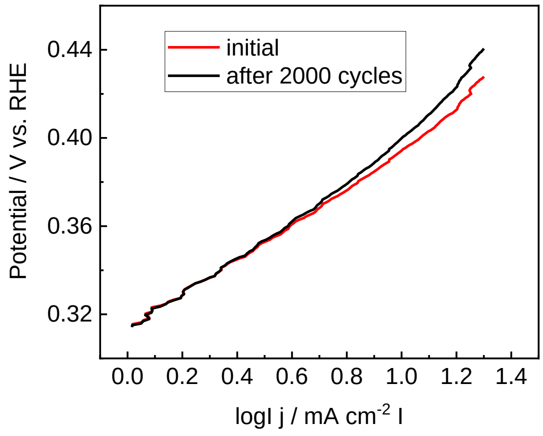

Figure S17 Tafel slopes of Ir-SA/NC electrocatalyst before and after 2000 cycles.

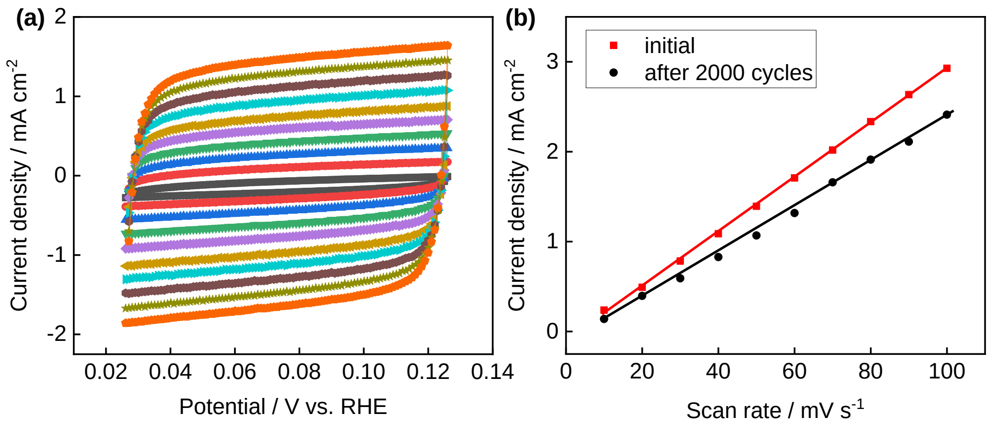

Figure S18 (a) Cyclic voltammetry curve of Ir-SA/NC after 2000 cycles. (b) C_{dl} of Ir-SA/NC before and after 2000 cycles.

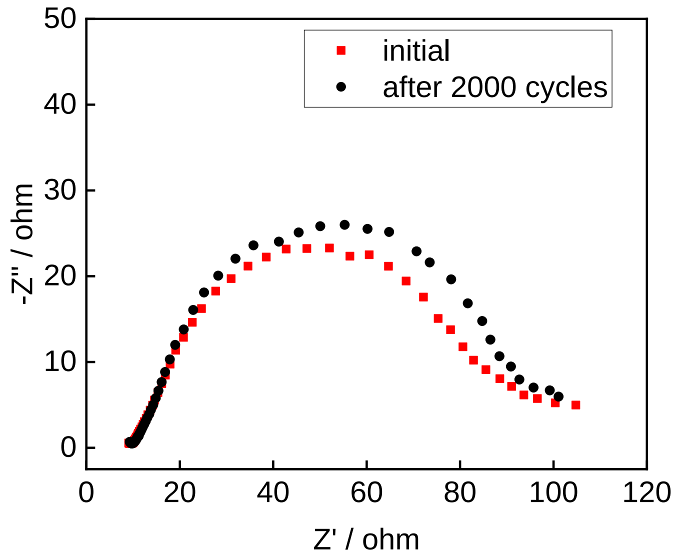

Figure S19 Electrochemical impedance spectroscopies of Ir-SA/NC before and after 2000 potential cycles.

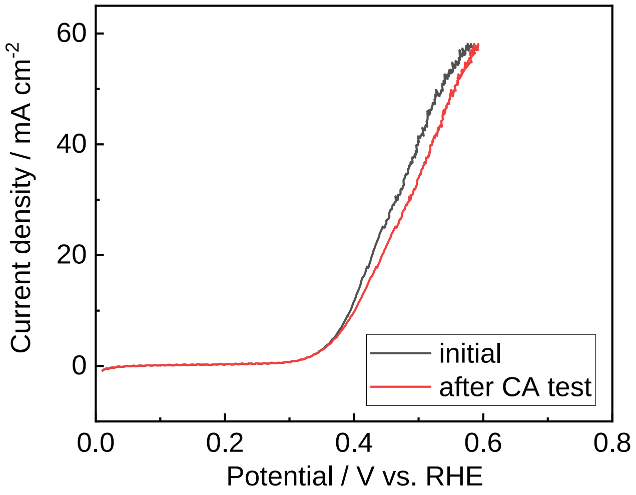

Figure S20 HzOR performance of Ir-SA/NC electrocatalyst before and after CA test.

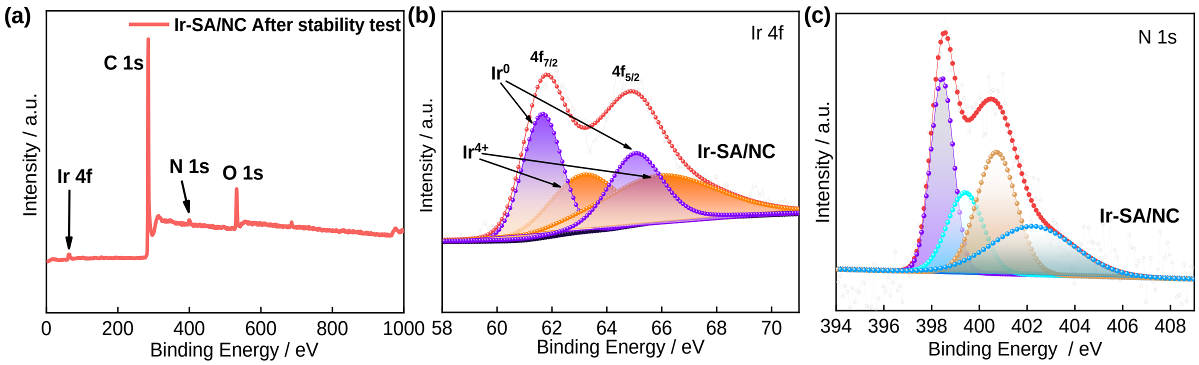

Figure S21 XPS survey scan (a), Ir 4f (b) and N 1s (c) of Ir-SA/NC after CA test.

References

[S1]H. Zhang, W. Wang, Z. Dai, Y. Zhu, M. Cheng, B. Zhang, Y. Feng, Y. Zhang, G. Zhang, J. Mater. Chem. A 2023, 11, 14674.

[S2]S. Zhao, Y. Zhang, H. Li, S. Zeng, R. Li, Q. Yao, H. Chen, Y. Zheng, K. Qu, J. Mater. Chem. A 2023, 11, 13783.

[S3]J. Li, C. Zhang, C. Zhang, H. Ma, Y. Yang, Z. Guo, Y. Wang, H. Ma, Chem. Eng. J. 2022, 430, 132953.

[S4]J. Wang, X. Guan, H. Li, S. Zeng, R. Li, Q. Yao, H. Chen, Y. Zheng, K. Qu, Nano Energy 2022, 100, 107467.

[S5]M. Zhang, Z. Wang, Z. Duan, S. Wang, Y. Xu, X. Li, L. Wang, H. Wang, J. Mater. Chem. A 2021, 9, 18323.

[S6]X. Guan, Q. Wu, H. Li, S. Zeng, Q. Yao, R. Li, H. Chen, Y. Zheng, K. Qu, Appl. Catal. B 2023, 323, 122145.

[S7]H.-L. Huang, X. Guan, H. Li, R. Li, R. Li, S. Zeng, S. Tao, Q. Yao, H. Chen, K. Qu, Chem. Commun. 2022, 58, 2347.

[S8]Q. Yang, B. Zhu, F. Wang, C. Zhang, J. Cai, P. Jin, L. Feng, Nano Research 2022, 15, 5134.

[S9]J. Shi, Q. Sun, W. Zhu, T. Cheng, F. Liao, M. Ma, J. Yang, H. Yang, Z. Fan, M. Shao, Chem. Eng. J. 2023, 463, 142385.

[S10] W. Tian, X. Zhang, Z. Wang, L. Cui, M. Li, Y. Xu, X. Li, L. Wang, H. Wang, Chem. Eng. J. 2022, 440, 135848.
